# Nanomechanical and surface properties of rMSCs post-exposure to CAP treated UHMWPE wear particles

**DOI:** 10.1016/j.nano.2015.10.006

**Published:** 2016-04

**Authors:** Emily Callard Preedy, Stefano Perni, Polina Prokopovich

**Affiliations:** a School of Pharmacy and Pharmaceutical Sciences, Cardiff University, Cardiff, UK; b Department of Biological Engineering, MA Institute of Technology, Cambridge, MA, USA

**Keywords:** rMSC, UHMWPE, Wear debris, AFM, Cold gas plasma

## Abstract

Wear debris generated by ultra-high molecular weight polyethylene (UHMWPE) used in joint replacement devices has been of concern due to reductions of the implant longevity. Cold atmospheric plasma (CAP) has been used to improve the wear performance of UHMWPE. Our aim was to investigate the elastic and adhesive properties of rat mesenchymal stem cells (rMSCs), through AFM, after exposure to UHMWPE wear debris pre- and post-CAP treatment. The results indicated that the main changes in cell elasticity and spring constant of MSC exposed to wear particles occurred in the first 24 h of contact and the particle concentration from 0.5 to 50 mg/l did not play a significant role. For UHMWPE treated for 7.5 min, with progression of the wear simulation the results of the CAP treated samples were getting closer to the result of untreated samples; while with longer CAP treatment this was not observed.

**From the Clinical Editor:**

Joint replacements are now common clinical practice. However, the use of ultra-high molecular weight polyethylene (UHMWPE) still poses a concern, due to the presence of wear debris. The authors here investigated the effects of wear debris after cold atmospheric plasma treatment on rat mesenchymal stem cells. The positive results provided new strategies in future design of joint replacement materials.

## Graphical abstract

Our paper describes for the first time the nanomechanical changes of mesenchymal stem cells after exposure to wear debris from UHMWPE untreated and treated with cold gas plasma that we previously showed to be capable of improving wear performance.

These changes help in understanding the mechanism involved in the response of stem cells to these debris that often lead to device failure with catastrophic results for the patients. Furthermore, they help identify the lack of cytotoxicity of the material after the cold gas plasma treatment.

**Figure:**
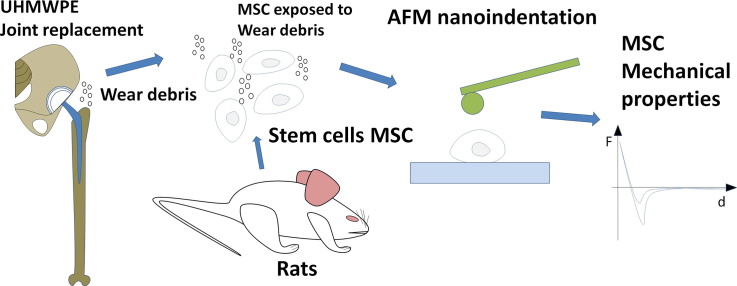

Orthopedic surgical procedures can replace the whole joint, or commonly the damaged or diseased sections may be replaced with a biomedical prosthesis often including articulating surfaces. Over time, the implanted devices are subjected to everyday stresses and strains encountered through simple movements and wear debris are inherently produced.^1^, ^2^, ^3^, ^4^ It has been noted that wear debris is produced regardless of the original material used in the implanted devices as it is a result of contacting surfaces rubbing and sliding against each other.^2^, ^4^, ^5^

UHMWPE is prone to generate wear particles after prolonged use^1^, ^6^, ^7^; debris with a range of diameters between 100 and 500 nm were observed, and some as large as 1-2 μm.^8^, ^9^, ^10^ The effect of wear particles on osteoblast cells can cause the cells to differentiate and prevent normal cell synthesis, thereby inhibiting the formation of new bone which in turn increases the resorption of the bone matrix inherently affecting osteoclast cells.^11^, ^12^, ^13^ It has been suggested that submicron UHMWPE particles (mean diameter smaller than 0.5 μm) greatly increase the induction of osteolysis than metal particles of lesser dimensions (diameter < 0.1 μm).^14^, ^15^, ^16^ More recently, only UHMWPEs larger than 50 nm have been shown capable of inducing osteolytic cytokine release from primary human peripheral blood mononuclear cells.^17^

Bone resorption, or osteolysis is the main culprit for future fracture and aseptic loosening of the surrounding bone and articulating surfaces, respectively; often resulting in revised surgery and replacement of the biomedical implant.

Certain treatments have been developed to improve the performance and modify the physicochemical properties of UHMWPE to highly cross-linked polyethylene (XLPE) employing rapid heavy ion beam irradiation and argon plasma treatment.^18^, ^19^, ^20^, ^21^, ^22^ Presently, UHMWPE is the choice material for articulating surfaces due to its promising properties despite its obvious flaws; yet a lack of *in-vivo* studies and production costs have limited the availability for the exploration of new and/or improved materials to be investigated as replacements for this polymer.^5^, ^6^, ^18^, ^21^, ^22^

In the last two decades, non-thermal plasma approaches have been used for the sterilization of heat sensitive materials and dental bleaching, respectively.^6^, ^23^ Cold atmospheric plasma (CAP) is produced at atmospheric pressure and is a partly ionized gas which has been more recently been applied to many medical technologies including wound cleaning, as well as surface modification for biomedical prosthesis.^6^, ^24^, ^25^, ^26^, ^27^ CAP is a beneficial technique compared to the more traditional thermal procedures due to the ease of scale up, simple operational set up, and cost effectiveness as there is no need for a vacuum chamber or heating appliances; in addition the process can be applied contact free acting as a sterilization technique as a result of its broad antimicrobial activity.^6^, ^23^ Positive results have been evident for the application of CAP treated material and its potency investigated on UHMWPE and metal surfaces reducing the generation of wear debris.^6^, ^28^, ^29^ CAP treatment has successfully been used to improve the wear performance of UHMWPE, reducing the wear rate by up to half after only 7 min of CAP treatment^6^ through increased cross-linking without detrimental effect on crystallinity. Additionally, the nitrogen group grafted on the material during CAP treatment increases the hydrophylicity and adhesion; while no oxygen was detected on the material after exposure to cold gas plasma.^6^, ^27^

Although CAP treatment has proven its reliability in its application to sterilize and strengthen UHMWPE, no investigation has yet delved into its affects toward the surrounding tissues and cells of an implanted biomedical device. Fundamentally, the following investigation is set to explore untreated and CAP treated UHMWPE wear debris potential impact on the nanomechanical and adhesive properties of rat mesenchymal stem cells. Employing atomic force microscope (AFM) in order to understand the impact of these debris on such properties and to determine whether CAP treatment has a potential detrimental effect that would preclude the use of this technology.

## Methods

### Polymer

Ultra-high molecular weight polyethylene (UHMWPE) cross-linked (4 Mrad) sourced from Germany (GUR 1020, Hoechst).

### Cold atmospheric plasma (CAP) source and UHMWPE treatment

**Fig. 1:**
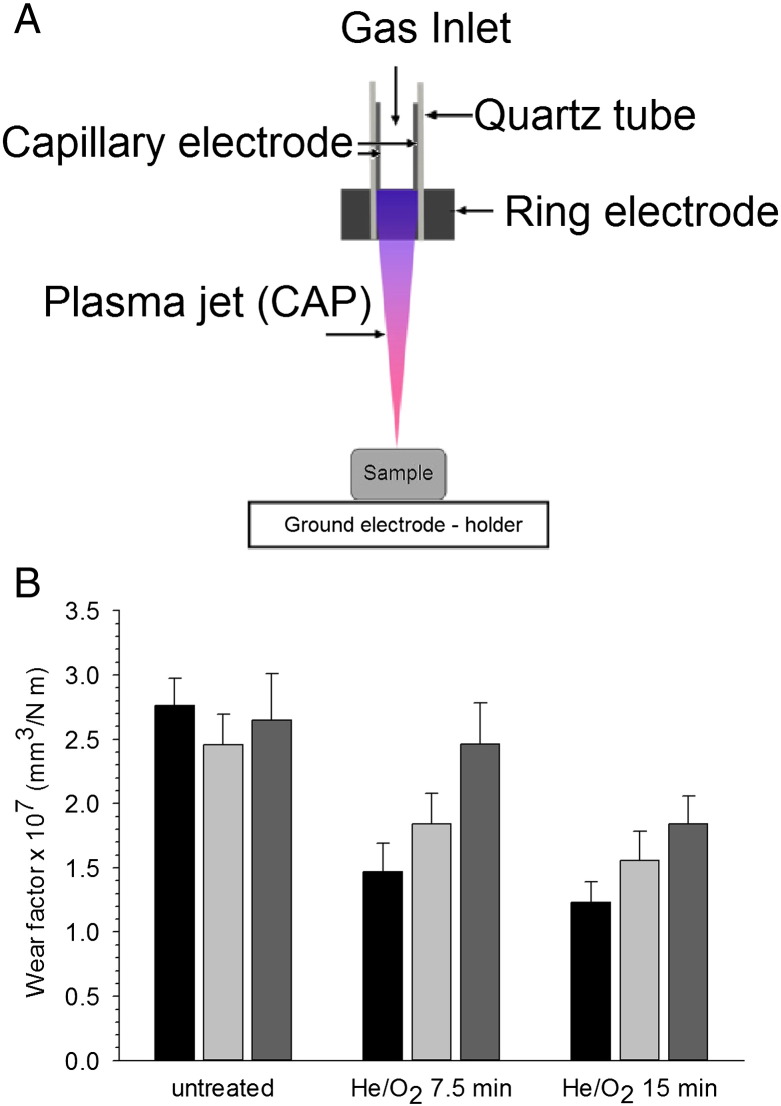
CAP equipment set up (a) and wear factors of treated and untreated UHMWPE (b).  333 kC; 333 kC–666 kC; 666 kC–1 MC.

The equipment used (Figure 1A) consisted of two main electrodes: a capillary and ring electrode. The capillary electrode is situated within the quartz tube with an inner diameter of 1.5 mm; downstream of this the ring electrode is wrapped around the quartz tube near the nozzle where the plasma jet is emitted. An axially directed electrical field is produced when an external voltage is applied as a result of the axial separation of the electrodes and due to the gas being introduced in an axial direction.^6^, ^24^ Helium and oxygen were mixed before entering the capillary tube with 5 slm (standard liter per minute) of 99.99% helium, and 10 sccm (standard cubic centimetre per minute) of oxygen. An alternating voltage supply, at a peak voltage of 8 kV and excitation frequency of 20 kHz, was applied to the gas mixture entering the capillary electrode in the quartz tubes causing an electrical discharge visualised as a light-emitting plume from the nozzle^6^, ^24^ CAP jet). Under the experimental conditions considered in this study the plasma plume was more than 1 cm long and its gas temperature was always below 27 °C. Samples to be treated were placed on a sample holder which was grounded electrically and the sample was fixed at 1 cm directly downstream of the quartz nozzle. The holder was rotated so that the entire UHMWPE was exposed to the CAP jet plume in turn. The samples were exposed to the plasma plume for 7.5 and 15 min. The treated samples were kept at room temperature and subjected to material and surface characterization within 1 day. Wear testing was initiated at the same time internal.

### Wear testing

Wear testing of UHMWPE was performed using a single station pin on plate in-house built wear simulator under constant load applied and under lubricated conditions. Pins were machined from UHMWPE while metallic plates were made of medical grade wrought cobalt–chromium alloy (according to ASTM F1537) and polished to an average surface roughness Ra 0.01 μm. Before wear testing all samples were soaked in distilled water at room temperature for 2 weeks, to saturate them. The lubricant employed in all wear tests consisted of 25 % v/v bovine serum (Harlan Sera-Lab, Loughborough, UK) in sterile water with 0.1% (w/v) sodium azide to inhibit the growth of bacteria. The lubricant was changed every week and serum samples were collected for wear debris analysis. Control pins were soaked in lubricant for the duration of the wear test and were used as a reference for mass change due to water uptake by the UHMWPE. The wear test was performed with multidirectional motion, the pins rotating 30° every 15 mm, and a sliding distance of 60 mm, resulting in a total pin rotation of 120°. The test cycle frequency was 1 Hz. A compressive load of 160 N was applied to an 8 mm diameter area of the pin surface, resulting in a nominal contact pressure of approximately 3.18 MPa. These testing conditions were selected to match the physiological range of contact pressures and lubrication found in human articular joints.^30^ At least six replicates were obtained for each type of plasma surface modification of UHMWPE.

The serum with wear particles was collected after 333 k cycles, 666 k cycles and 1 M cycles and particles extracted.

The mass loss of the polymer due to wear process was obtained gravimetrically taking into account the mass change due to the moisture absorption of control pins. The wear factor (*W*) was calculated as the volume loss per unit load per unit sliding distance as:

(1) $$W=\frac{V}{F}L$$

where *V* is volume loss during wear phase; *L* is the sliding distance; *F* is the compressive load.

### Isolation of UHMWPE wear debris from serum

From the lubricant, bovine serum, used in the wear simulator test, the wear particles are isolated by adding 2 g of KOH to 100 ml of the bovine serum with wear debris. This suspension was placed in a water bath at 60 °C with continuous stirring for 48 h; the solution was removed and allowed to cool to room temperature before placing in the fridge for 30 min to reduce the temperature to 4 °C. Once cool, a 10 ml mixture of chloroform:methanol (2:1) was added to the solution and incubated in a fume cupboard at room temperature for 24 h. The suspension was then centrifuged at 2000 rpm at room temperature to remove unwanted proteins and lipids contained in the lubricant for 20 min; the supernatant was removed and the process was repeated with the addition of the chloroform:methanol mixture until the supernatant was clear and all proteins and lipids removed. Following the centrifugation of the samples, filtration was required using a Buchner filter and 0.2 μm filter membranes (Whatman, UK), to collect all wear debris remaining in the supernatant solution. The filters were air dried in a fume cupboard overnight in sterile covered petri dishes. All filters membranes were weighed before and after filtration to determine the yield of wear debris.

### Wear debris analysis

#### SEM analysis

The extracted UHMWPE wear debris on the membrane filters were gold coated and SEM images were acquired. Scanning electron microscopy was performed in a Hitachi filament scanning microscope, using a filament voltage of 5 keV. A minimum of 15 images at random locations were acquired for each filter in secondary electron mode. Wear debris were identified from the images and the shape and size individually determined. This process was performed individually on serum obtained from each of the replicates of the various treated and untreated materials and the results are presented as overall means ± SD.

#### Raman analysis

Raman spectra were obtained using a Renishaw Raman System 1000 (Renishaw plc) with an Ar + laser (514 nm). The instrument was fitted with an external Olympus BH-2 microscope and the spectra were collected using a personal computer.

#### XRD analysis

X-ray diffraction (XRD) analysis was performed using a Bruker-Axs D8 (GADDS) diffractometer, utilizing a large two-dimensional area detector a monochromatic Cu X-ray source (Ka1 Ka2) fitted with a Gobble mirror. The instrumental set-up gave 34° for both *h* and *x*, with a resolution of 0.01° 3-4 mm^2^ of sample surface illuminated at any one time. Multiple Debye–Scherrer cones were recorded simultaneously by the area detector with two sections covering a 2 h range of 65°. The Debye–Scherrer cones were integrated along *x* to produce standard one dimensional diffraction patterns of 2 h against intensity. Scan data were collected for 800 s to give sufficiently resolved peaks for indexing.

### Cell culture

28-day-old, male Wistar rats were obtained from the colony maintained by Charles River European Suppliers (Charles River UK Ltd., Kent, UK). The animals were housed with free access to water and were maintained with treatment and care protocols conformed to UK Animals (Scientific Procedures) Act 1986, accordance to the European Convention for the Protection of Vertebrate Animals Used for Experimental and Other Scientific Purposes (Strasbourg, Council of Europe). Bone marrow stem cells were isolated from rat femur and humerus, using plastic adherence,^31^ followed by fibronectin adherence techniques.^32^ After 7 days, merged colonies were expanded (passage 0). This study was conducted on cells obtained from early population doubling level.

The cells were routinely cultured in α-MEM (Life Technologies), supplemented with 20% (v/v) FBS, 1% (v/v) of solution penicillin (5000 U/mL) and streptomycin (5000 mg/mL) (Gibco Invitrogen) and 1% (v/v) of L-ascorbic acid 2-phosphate solution at 50 mg/ml (Sigma, UK). Accutase (Gibco Invitrogen) was used when cells were 70% confluent in order to passage and count. The cells were maintained at 37 °C in a humidified atmosphere containing 5% CO_2_.

For atomic force microscopy experiments, cells were seeded in 24-well plates at a density of 6000 cells per well and cultured for 24 h on sterilized polystyrene slides placed inside the well before exposure to UHMWPE wear debris. For each type of the wear debris sample a stock solution of UHMWPE particles suspended in culture media was prepared at 5 mg/ml and appropriate amount was added to each well to reach final concentrations of 5; 25 and 50 μg/ml and incubated from 24 h up to 3 days. Control samples consisting of cells not exposed to the wear particles and cultured in the same conditions were used for comparison with treated cells.

For the MTT assay, the cells were cultured as described above.

### Nanomechanical and adhesive properties of rMSCs measurements

All AFM force measurements were conducted with an Advanced Scanning Probe Microscope (XE-100, Park Systems, Korea) in an open liquid cell as described in ^27^ using PBS as the aqueous phase. A triangular tipless cantilevers (Bruker, UK) and a nominal spring constants (*K*_cantilever_) of 0.1 N/m were used; the actual spring constant of the AFM cantilever was determined using the Sader method.^33^, ^34^ Borosilicate glass beads (10 μm in diameter) were glued onto the cantilever and served as cell indentor. In order to prevent indentations depth greater than 400-500 nm, the maximum applied load was set, after preliminary tests, to 1 nN or 2 nN depending on the samples. At least 15 cells were analyzed for each sample, at each concentration (0, 5, 25, and 50 μg/ml) and at each time point (24, 48, and 72 h). Cells were first located and then at least 20 approaching and retracting z-piezo coordinates vs. deflection curves were extracted from randomly selected points on the surface of each cell avoiding the peri-nuclear region. Experiments were performed in triplicates.

#### Cell elasticity and cell spring constant determination

The approaching part (trace) of the AFM curves was used to calculate the nanomechanical properties of the cells. The Young modulus of the cell surface location under investigation was determined fitting the Hertz model (Eq. (2)) to the first part of the indentation vs. force curve after contact between AFM tip and cell surface.

(2) F=43E1‐ν2Rδ23

where:

*F*: force recorded by AFM
*E*: Young modulus
*R*: radius of the spherical indentor (5 μm)
*ν*: Poisson ratio (set at 0.5)
*δ*: indentation depth

The spring constant of the cell surface in the location probed was determined through the slope of the curve after the Hertzian regime according to:

(3) $$F=\;k_{b}\delta$$

where:

*F*: force recorded by AFM
*K_b_*: spring constant of the cell
*δ*: indentation depth

Both models require the determination of the separation between cell surface and AFM tip (*δ*), this was calculated from the coordinates (z-piezo) of the trace curve assuming that the point of contact corresponded to the local minimum of force; from this:

(4) $$\delta =\left|z-z_{0}\right|-d_{\mathrm{cant}}$$

where:

*z_0_*: z-piezo value of the minimum of the trace curve
*z*: z-piezo value of the trace curve
*d*_cant_: cantilever deflection
*δ*: indentation depth

and

(5) $$F=K_{\mathrm{Cantilever}}d_{\mathrm{cant}}$$

Both Eqs. (2), (3) were fitted to the data using the least squares method through an in-house written FORTRAN code.

#### Cell adhesion force

The adhesion forces between a cell and AFM tip were determined as the minimum value of the retracting (retrace) part of the AFM curve.

### Metabolic activity assay

MTT (3-(4,5-dimethythiazol-2yl)-2,5-diphenyltetrazolium bromide) assay was used to determine the effects of the metal nanoparticles on MSCs viability. Cells were initially cultured and exposed to nanoparticles as stated above in a 24-well plate; after the chosen exposure time to the wear particles, the media was replaced with phenol red-free medium and 80 μl of MTT stock solution (5 mg/ml) was added to each well and incubated at 37 °C in humidified atmosphere containing 5% CO_2_ for 2 h. The metabolized MTT, formazan, was re-suspended with 800 μl of dimethyl sulfoxide (DMSO). 200 μl was transferred to a 96-well plate absorbance at 560 nm which was read using a spectrophotometer (ELISA Reader Labtech LT-5000MS). All experiments were performed in triplicates with each concentration (5, 25 and 50 μg/ml) as well as a control sample of cell suspension not exposed to the wear particles (untreated cells).

### Statistical analysis

Because of their normal distribution, both metabolic, elasticity and spring constant data were analyzed using one-way ANOVA to determine any significant difference between the mean values of the concentrations used this was followed by Tukey's post-hoc test (*P* < 0.05). The not normally distributed adhesion forces were compared using the Kruskal–Wallis test followed *post hoc* with a Dunn's test for individual pairs of data sets. Statistical analysis was performed using SPSS.

## Results

### Wear characterization

The wear rate of untreated UHMWPE did not change with wear progression (Figure 1, B) (*P* > 0.05), while both CAP treated samples exhibited lower wear rates than untreated materials after 333 kC and between 333 kC and 666 kC (*P* < 0.05). During the wear period between 666 kC and 1 MC the samples CAP treated for 7.5 min had the same wear factors as untreated UHMWPE (P > 0.05), on the contrary 15 min CAP treated samples still had lower wear factors than untreated samples (*P* < 0.05).

**Figure 2:**
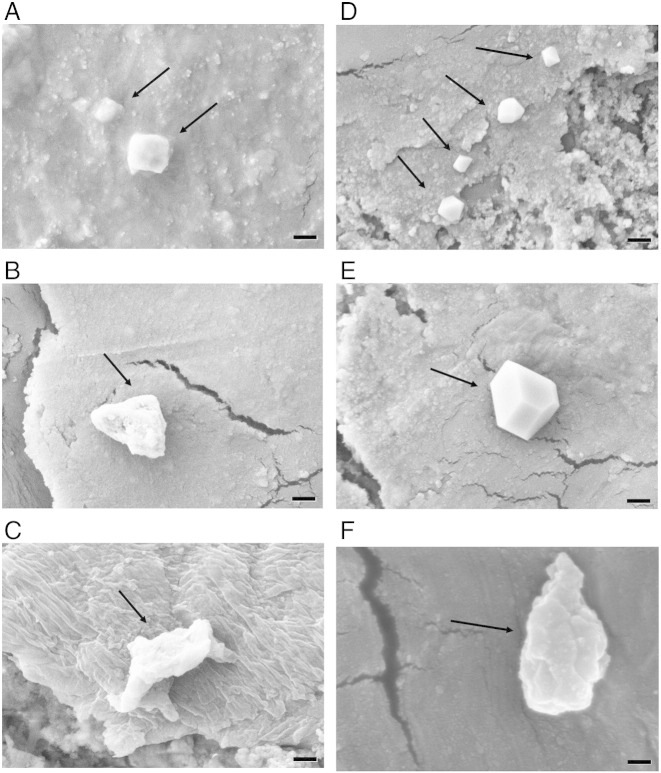
SEM of wear debris from untreated UHMWPE after 333 kC (**A**), 666 kC (**B**) and 1 MC (**C**) and from 15 min CAP treated UHMWPE after 333 kC (**D**), 666 kC (**E**) and 1 MC (**F**). Bar represents 400 nm and arrows indicate debris.

**Fig. 3:**
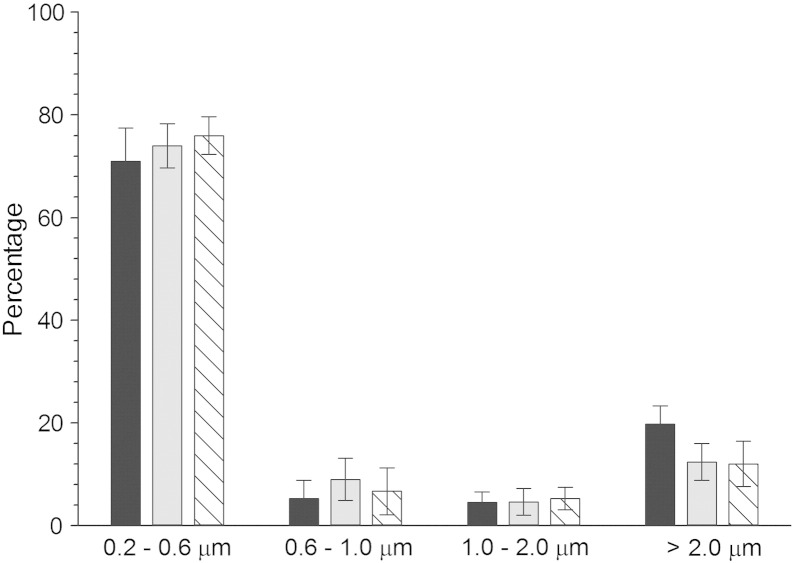
Size distribution of the wear debris produced after wear simulation of UHMWPE.  untreated UHMWPE;  7.5 min treated UHMWPE;  15 min treated UHMWPE.

Scanning electron microscope was used to determine size and shape of the UHMWPE wear particles. The UHMWPE debris produced during wear simulation, extracted from the lubricant, were generally circular in shape with some elongated regardless of the CAP treatment, examples are shown in Figure 2; debris of different geometry (prism in Figure 2, A, D and E; globular in Figure 2, B, C and F) and roughness have been found (globular generally rougher than prism). From the size distribution data presented in Figure 3, it is evident that the great majority (around 70%) of debris had a diameter between 0.2 and 0.6 μm, while a small proportion of the debris was between 0.6 and 2 μm, and about 15% of the debris was larger than 2 μm. It is also clear that the CAP treatment had no influence in the size distribution of the wear debris.

**Figure 4:**
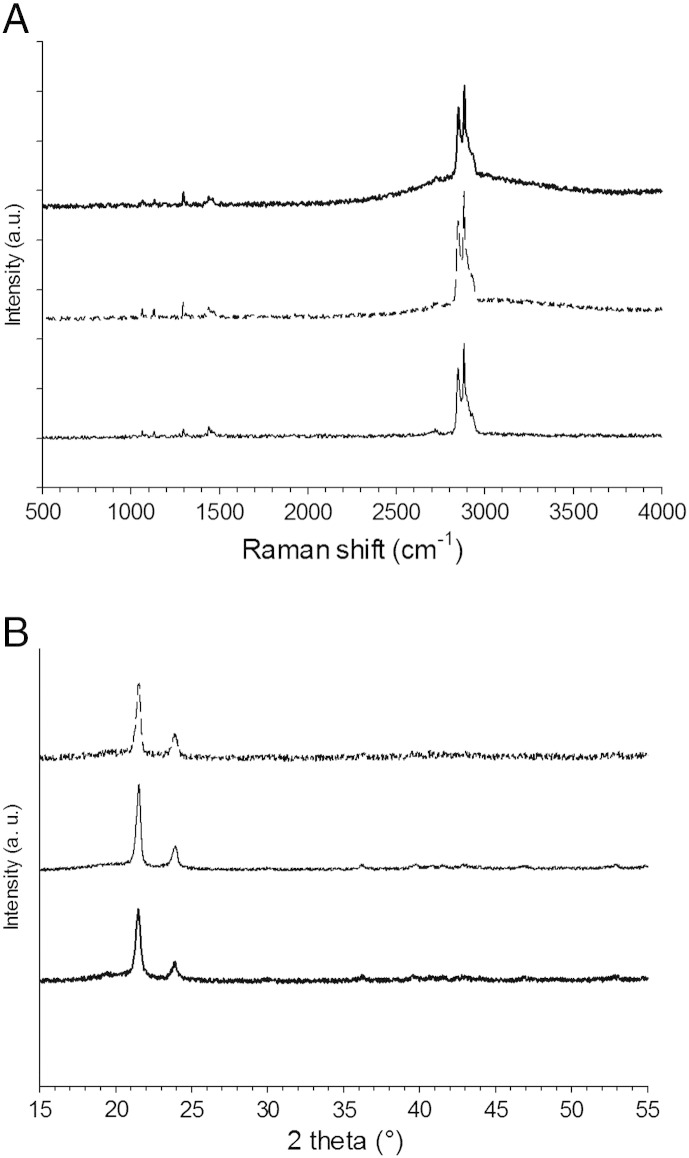
Raman spectra (**A**) and XRD diffraction patterns (**B**) of untreated UHMWPE (thick solid line) and treated with cold gas plasma for 7.5 (thin solid line) and 15 min (dashed line).

Raman spectroscopy was employed to investigate the composition of the surface of the samples after treatment. The spectra of the surface of UHMWPE (Figure 4, A) showed two high peaks at about 2800 cm^− 1^ that are typical of the bond between carbon and hydrogen. In the range 1000-1500 cm^− 1^ four small peaks were evident which are typical of UHMWPE. The bands at 1080 and 1127 cm^− 1^ are due C—C stretching, the band at 1293 cm^− 1^ to twisting of crystalline –CH2–, the band at 1365 cm^− 1^ is related to amorphous C—C twisting and that at 1440 cm^− 1^ to the bending of crystalline C—C, while peaks in the regions between 2400 and 2800 and from 3000 to 3300 cm^− 1^ can be attributed to nitrogen compounds.^35^ After 7.5 min of cold plasma treatment the Raman spectra presented a wide peak at a Raman shift between 3000 and 3300 cm^− 1^, which is characteristic of the bond between carbon and nitrogen. A similar peak was also evident in the samples exposed to cold gas plasma for 15 min. The region between 2400 and 2800 cm^− 1^ that was altered in the samples treated for 7.5 and 15 min can also be attributed to nitrogen compounds.

There was no noticeable difference in terms of the XRD diffraction patterns (Figure 4, B) with and without plasma treatment, demonstrating that the cold atmospheric pressure plasma did not affect the crystallinity of UHMWPE.

### Metabolic activity

**Fig. 5:**
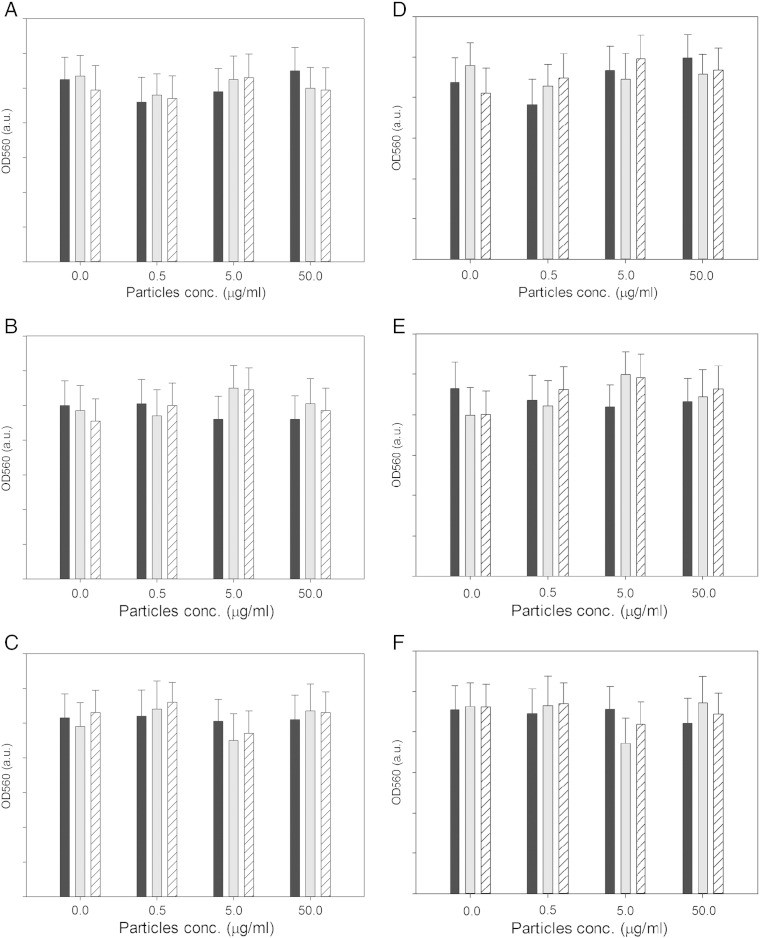
MTT results of MSCs exposed for 24 h to UHMWPE wear particles after 333 kC (a), 666 kC (b) and 1 MC (c) and for 72 h to UHMWPE wear particles after 333 kC (d), 666 kC (e) and 1 MC (f).  untreated UHMWPE;  7.5 min treated UHMWPE;15 min treated UHMWPE.

The metabolic activity of osteoblast cells exposed to wear particles of untreated UHMWPE or after CAP treatment did not change with the particles concentration, number of wear cycles and exposure time (Figure 5). Furthermore, the CAP treatment had no consequence on the metabolic activity of the cells (*P* > 0.05).

### Elasticity, spring constant and adhesion

**Fig. 6:**
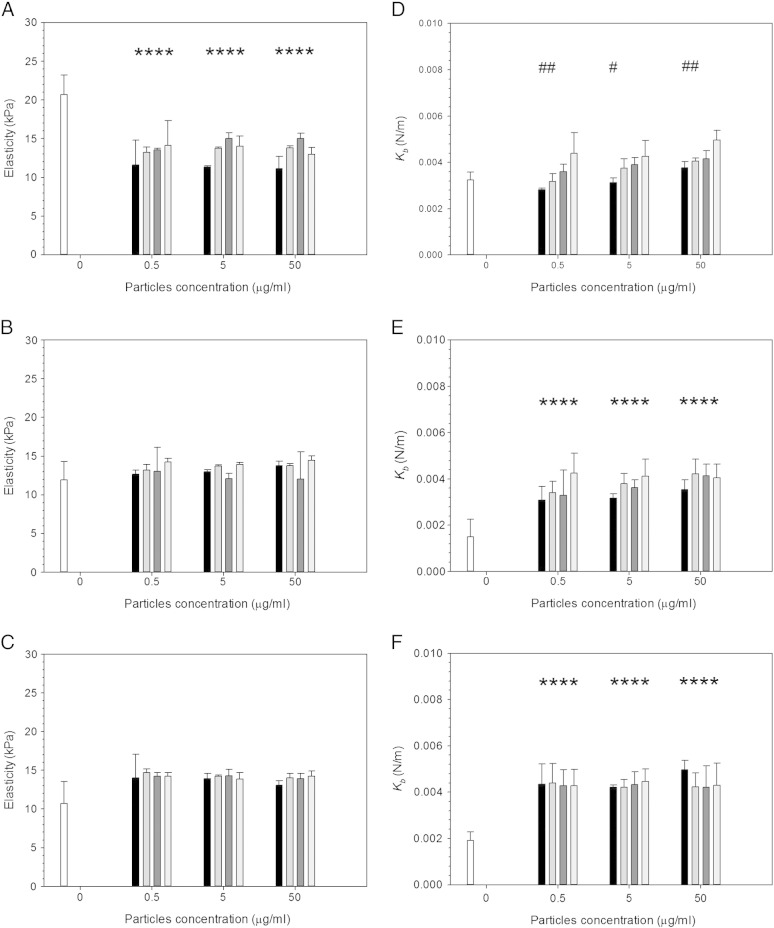
Mean cell elasticity (left) and spring constant (right) of MSCs exposed to UHMWPE wear particles pre- and post- CAP treatment for 7.5 min for (a, d) 24 h, (b, e) 48 h and (c, f) 72 h.  Control;  UHMWPE 333 kC; UHMWPE 666 kC; UHMWPE 1 MC;  untreated UHMWPE. * represents samples statistically different from samples not exposed to any particle. # represents samples statistically different from samples exposed to debris originated from untreated UHMWPE.

**Fig. 7:**
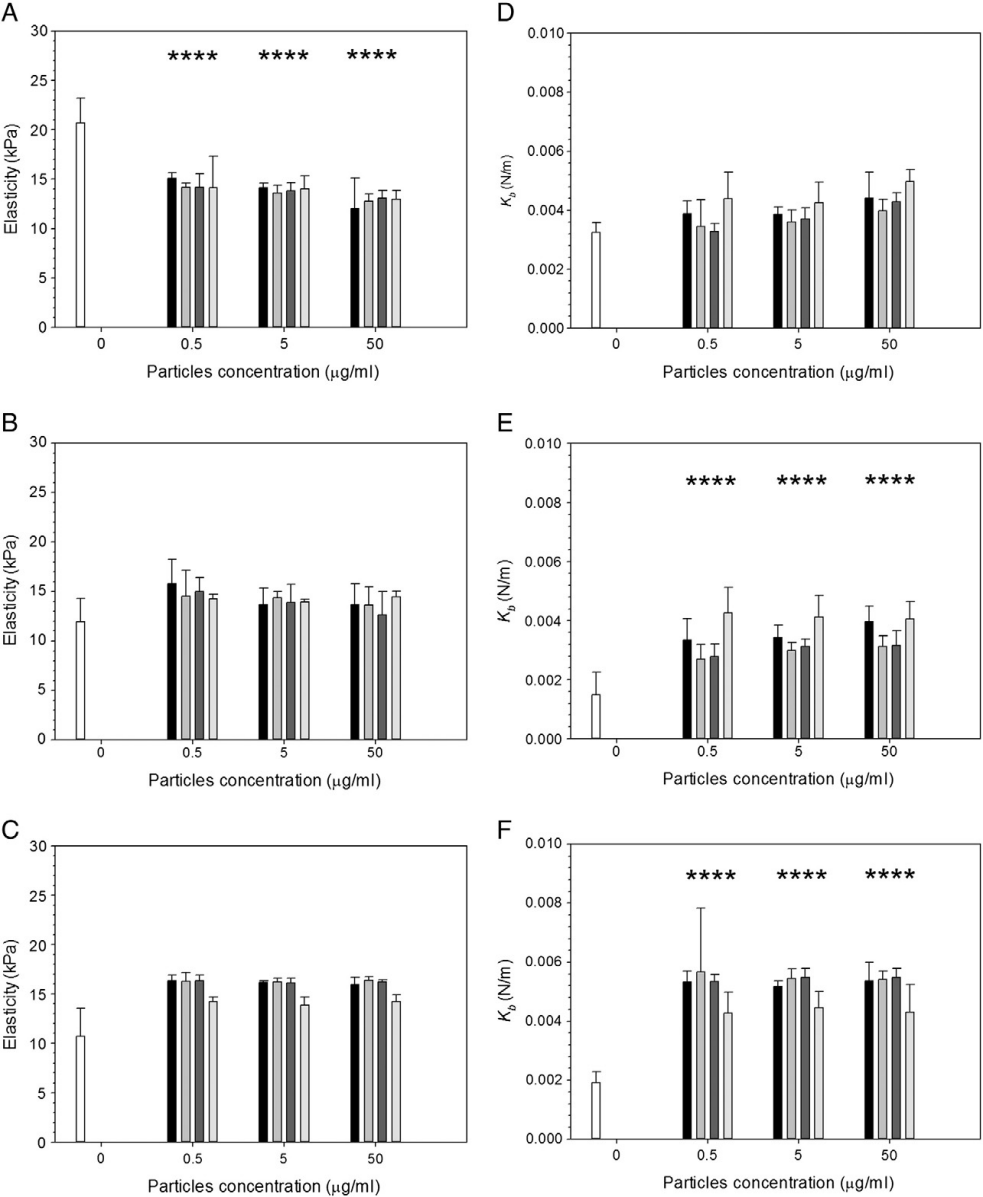
Mean cell elasticity (left) and spring constant (right) of MSCs exposed to UHMWPE wear particles pre- and post- CAP treatment for 15 min and for (a, d) 24 h, (b, e) 48 h and (c, f) 72 h.  Control;  UHMWPE 333 kC; UHMWPE 666 kC; UHMWPE 1 MC;  untreated UHMWPE. * represents samples statistically different from samples not exposed to any particle.

Elasticity measurements were conducted using the AFM over 3 days. Cells were exposed to wear debris over time at different concentrations ranging from 0.5 to 50 μg/ml, and 9 types of UHMWPE debris were used, untreated UHMWPE along with 333 kC, 666 kC and 1 MC CAP treated for either 7.5 min (Figure 6) or 15 min (Figure 7).

For cells exposed to untreated samples of UHMWPE (Figure 6) elasticity was lower compared with the control after 24 h exposure (*P* < 0.05), while no difference in elasticity was noted after 48 h and 72 h exposure and no effect of concentration was demonstrated (*P* > 0.05). For cells exposed to 7.5 min CAP treated samples, (Figure 6), a reduction in elasticity was recorded after 24 h exposure compared to cell not exposed to any particle (*P* < 0.05), while no difference was noted after 48 h and 72 h compared with control cell in both cases (*P* > 0.05). Moreover, in all cases no effect of concentration was detected (*P* > 0.05). Wear progression (333 kC, 666 kC and 1 MC) resulted in increasing elasticity only for the shortest exposure time (*P* < 0.05).

For cells exposed to 15 min CAP treated samples, Figure 7, a decrease of elasticity was observed (*P* < 0.05) after 24 h compared to control samples (cells not exposed to any particle). On the other hand, an increase of elasticity was seen after 72 h exposure compared to control cells (*P* < 0.05). No effect was observed on cells elasticity from varying concentration of wear particles or wear rate (*P* > 0.05).

Both 7.5 min and 15 min CAP treatments had not significant impact of UHMWPE debris caused on the cell elasticity as not difference compared to untreated samples was detected (*P* > 0.05)

For the spring constant values, untreated UHMWPE samples, no change was observed for increasing concentration or with exposure time, all spring constant values for cells exposed to the wear debris had a value of around 0.004 N/m (*P* > 0.05). However, this was greater than the resulting values for cells not exposed to any particle (*P* > 0.05). Additionally, cell not exposed to any particles exhibited a decreasing spring constant with longer culture time (*P* < 0.05).

For MSC cell exposed to 7.5 min CAP treated UHMWPE samples (Figure 6) the spring constant of the cells generally increased with increasing wear progression for 24 h exposure (*P* < 0.05). After 24 h, similar values for 7.5 min CAP treated and untreated UHMWPE were observed for cell exposed to wear particles originated after 1 MC (*P* > 0.05); furthermore, no significant effect of the wear particle concentration was detected (*P* > 0.05). After 48 and 72 h of exposure, no significant difference in cell spring constant was detected between cell exposed to debris obtained from untreated UHMWPE or CAP treated samples (*P* > 0.05); also the concentration of the particles, in the range tested, did not cause a significant effect on cell spring constant (*P* > 0.05).

Spring constant for cells exposed to wear debris of UHMWPE CAP treated for 15 min is shown in Figure 7. All samples (untreated and CAP treated) had values of around 0.0055 N/m without differences related to wear particle concentration or wear progression (*P* > 0.05), but in all cases significantly higher than cell not exposed to any UHMWPE debris (*P* < 0.05).

**Fig. 8:**
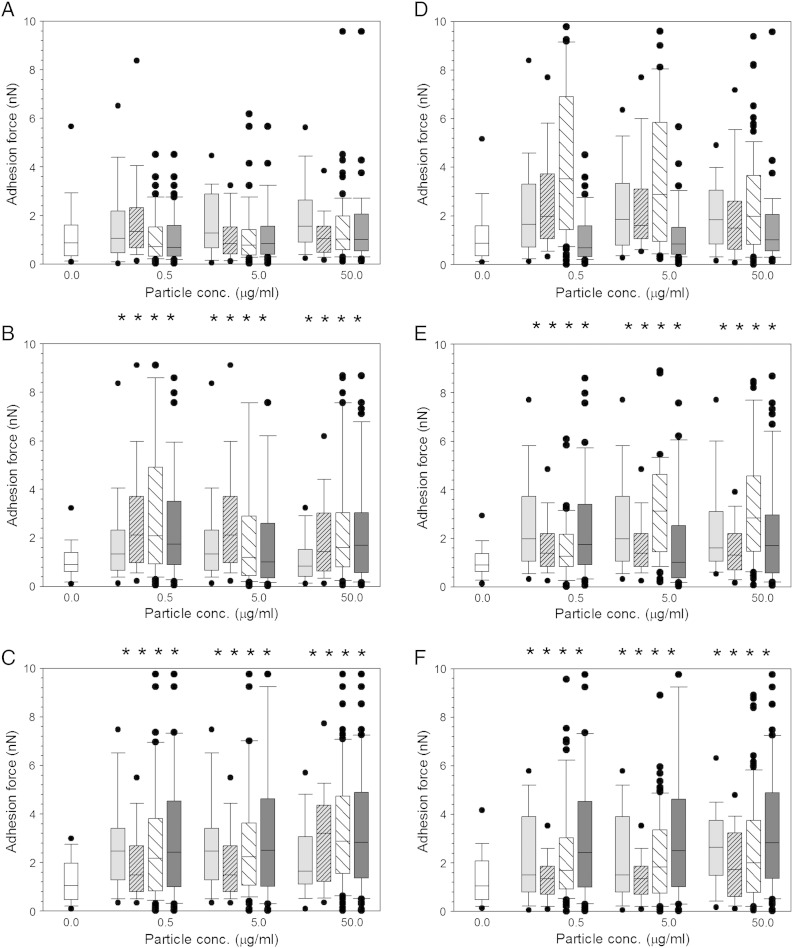
Adhesion force distribution of MSC cells exposed to UHMWPE wear particles pre- and post- CAP treatment for 7.5 min (left) and 15 min (right) and for (a, d) 24 h, (b, e) 48 h and (c, f) 72 h.  Control;  UHMWPE 333 kC;  UHMWPE 666 kC; UHMWPE 1 MC;  untreated UHMWPE. * represents samples statistically different from samples not exposed to any particle.

Another important property of the cells that was investigated was the adhesion characteristics of the cells pre and post-exposure to CAP treated and untreated wear particles (Figure 8), in all cases they did not follow a Gaussian distribution. For untreated UHMWPE the adhesion increased with exposure time (*P* < 0.05) and did not change for particle concentration in the range tested in this work (*P* > 0.05). Compared to cell not exposed to any particles the adhesion forces were higher for cell exposed to UHMWPE particles only after 48 and 72 h (*P* < 0.05) but not after 24 h (*P* > 0.05). After exposure for 24 h to 7.5 min CAP treated UHMWPE wear debris (Figure 8), at all concentrations tested, no change was observed when comparing the control cell or untreated UHMWPE (*P* > 0.05). After 48 h exposure to wear debris, an increase in adhesion forces was observed for all CAP treated UHMWPE when compared with the control cell samples (*P* < 0.05) but not when compared to untreated UHMWPE (*P* < 0.05). At 72 h exposure, a similar increase is demonstrated when compared with the control cells (*P* < 0.05), yet no change was observed with increasing concentration (*P* > 0.05) and no difference was noticed between treated and untreated samples (*P* < 0.05).

UHMWPE wear debris originated after 15 min CAP treatment, also demonstrated a similar pattern of increasing adhesion forces when compared to control samples and not difference between untreated and CAP treated samples after 48 h and 72 h exposure time (*P* > 0.05). In no case the particle concentration did cause significant differences in the adhesion forces measured (*P* > 0.05).

## Discussion

### CAP treated UHMWPE wear particles effect on rMSCs metabolic activity

The size distribution of the UHMWPE debris found in this work (Figure 3) is in line with the findings of other works that reported the majority of debris around 1 micron of globular, fibrillar and flake shape.^36^ The debris generated by CAP treated samples appear also smoother and with a prism shape that could be linked to different chemical properties of the treated materials. Because of the different chemical compositions of the wear debris after CAP treatment (the presence of nitrogen groups predominantly (Figure 4) along with the shape and roughness (Figure 2), it is important to determine the possible implication on the metabolism of mammalian cells of these debris. It has been shown that osteoblast cells can grow on UHMWPE unaffected by CAP treatment with He/oxygen mixtures.^6^ In this work, we determined the effect of the UHMWPE wear particles generated on the metabolic activity of MSC and found that these cells are fundamentally unaffected by UHMWPE at concentrations up to 50 mg/l and CAP treatment does not cause alternation of this (Figure 5). Poly-ethylene is a relative inert material as the result of the MTT test demonstrating that untreated UHMWPE at concentration up to 50 mg/l had the same results as cells not exposed to any wear particle. The added nitrogen groups by the CAP treatment do not cause variation of the level of UHMWPE cytotoxicity.

### Elasticity and spring constant of rMSCs exposed to CAP treated UHMWPE wear particles

Previously, CAP has been reported to improve the longevity of biomedical devices due to its reduction in the generation of wear by minimizing the asperities of the surface of UHMWPE^24^; the determination of the responses of cell when exposed to debris obtained from the treated material was performed in this work as, after exposure to CAP, practically a new material is produced.

AFM allows for ease of investigation as no sample preparation is required and cells can be imaged and probed in biological conditions minimizing damage and potential changes that may occur with other techniques that require fixation of cells.^37^, ^38^, ^39^ Previous work conducted on cells using AFM has suggested that an applied force ranging between 1 and 100 nN is sufficient to initiate cellular responses,^39^ this confirms the selection of a fixed applied load up to 2 nN for the above investigation. The low value of applied force is connected to a small indentation depth that is needed to prevent cell damage.

A general increase in adhesion properties of the cells was observed when exposed to wear debris; this could be due to wear debris interacting with the cells surface altering the overall adhesion mechanisms of the cells.

Elasticity of cells is an important aspect to consider in the normal function of a cell, and it is thought that wear debris may alter the usual differentiation of cells and can induce osteolysis which affects osteoblasts and osteoclasts.^2^, ^40^ Investigating the effects induced to MSCs is essential as these cells can differentiate into a variety of phenotypes including osteoblasts, cartilage, ligaments, adipocytes, muscle and connective tissue.^41^, ^42^ Owens and Solursh^43^ demonstrated that rat MSCs behave in a similar manner to human MSCs and are often used as an approximation to human cells. Another notion of elasticity is to consider it as the degree of deformation of a cell in response to an applied load; some studies have shown that a relationship exists between the elasticity of cells and their vital functions.^41^, ^44^ Not only do cells probed by an applied load are affected, but also surrounding molecules such as the extracellular matrix (ECM), transmembrane proteins, chromatin, cytoskeleton and lipid bilayer also respond to external as well as internal forces resulting in deformation of the structures of the cells in question^41^; therefore, the elastic properties of the MSCs have been measured to determine their effect of wear debris on the cells properties.

The model used to determine the elastic characteristics of the cells is based on the Hertz model, to apply this model seven assumptions must be fulfilled: (i) the material of the contacting bodies is isotropic and homogenous; (ii) the loads applied are static; (iii) the material is linearly elastic in nature; (iv) The curvature of radii of the contacting bodies are much larger than the contact radius; (v) the dimensions of the bodies are much larger than the dimensions of the contact surface; (vi) the contacting surfaces are smooth; and (vii) the deformations are small.^45^ As the geometry of the tip satisfies all the above points, the Hertz equation can be assumed,^46^ and all other known parameters including indentation depth, *δ*, and force, *F*, are determined using the AFM, and the Poisson ratio (when a material is compressed in one way and expanded in another direction perpendicular to the compression) is taken as 0.5 for soft elastic biological samples.^46^

Wear debris of UHMWPE have a significant impact on MSC elasticity (Figure 6). Without the presence of particles, cells exhibit a decreasing elasticity from about 20 kPa to about 10 kPa over the period from 1 to 3 days; when MSCs are exposed to UHMWPE debris the elasticity of the cell remains at about 15 kPa regardless of the debris concentration during the same exposure time. The most remarkable fact is the increasing elasticity of MSC exposed for 24 h to debris obtained from progressive wear (333 kC to 666 kC to 1 MC) of 7.5 min CAP treated UHMWPE at debris concentrations greater than 5 mg/l. The elasticity values obtained from the wear simulation between 666 kC and 1 MC return values closer to pure UHMWPE than the debris generated at the beginning (333 kC). This phenomenon is consistent with the fact that the outer layer of the treated samples is more likely to exhibit the effect of the CAP treatment as result of the decreasing penetration of the plasma species; thus the initial wear generated presents to the biggest modification induced. This hypothesis is also sustained by the observation that the longer CAP treatment of 15 min (that is resulting in material modification deeper in the samples) does not exhibit such behavior. It appears that the short treatment improves a thinner layer of UHMWPE; this is consistent with the wear factors shown in Figure 1, B.

Our study of the cell spring constant (monotonically linked to turgidity) revealed that the presence of UHMWPE causes the cell to exhibit higher pressure. Increased turgidity has been shown to be a mechanism involved regulating cell uptake and membrane trafficking.^47^ Elasticity and turgidity are linked as cell size can only change slightly as result of pressure variation, as swelling or shrinking is a threat to cell viability.^48^, ^49^ An increase in turgidity can be withstood with increased elasticity.

Our work demonstrates that debris, originated from UHMWPE routinely used in joint replacement devices, cause an impact on the mechanical properties of cells and that CAP treatment is a useful approach to improve the wear performance of UHMWPE as it does not alter the mechanical responses of cells exposed to debris originated from the wear process compared to untreated UHMWPE.
